# Normative data for handgrip strength in Iranian healthy children and adolescents aged 7–18 years: comparison with international norms

**DOI:** 10.1186/s13052-021-01113-5

**Published:** 2021-07-30

**Authors:** Sajjad Rostamzadeh, Mahnaz Saremi, Alireza Abouhossein, Shahram Vosoughi, Johan F. M. Molenbroek

**Affiliations:** 1 Occupational Health Research Center, Iran University of Medical Sciences, Tehran, Iran; 2 Workplace Health Promotion Research Center (WHPRC), School of Public Health and Safety, Shahid Beheshti University of Medical Sciences, Tehran, Iran; 3 Occupational Health Research Center, Department of Occupational Health Engineering, Faculty of Health, Iran University of Medical Science, Tehran, Iran; 4 Faculty of Industrial Design Engineering, Delft University of Technology, Delft, Netherlands

**Keywords:** Normative data, Handgrip strength, Children, Adolescents, Jamar dynamometer

## Abstract

**Background:**

Grip strength is an essential component of physical fitness. The objective of this study was to develop normative handgrip strength data for Iranian healthy boys and girls comparing their handgrip strength with international reference values.

**Methods:**

Handgrip strength was measured in 2637 healthy children/adolescents (1391 boys and 1246 girls), aged 7–18 years, using a standard adjustable Jamar hand dynamometer (Model 5030 J1, Sammons Preston Rolyan, Bolingbrook, IL, USA). Body mass (kg) and stature (cm) were measured and body mass index was computed in kg/m^2^. The sample was stratified by gender, age, and hand preference.

**Results:**

Handgrip strength increased with age and was considerably higher in boys than in girls for all age groups (*p* < 0.001). Grip strength had a parallel and linear growth for both genders until the age of about 11 years and showed a steeper upward slope in boys than in girls thereafter. The findings of the current investigation were significantly different from those of the previously published normative data, especially for boys over the age of 12 years and girls in the age range of 7–18 years (*p* < 0.001). This difference was mainly in such a way that the Iranians had lower handgrip strength.

**Conclusions:**

The differences between present results and those of similar available in the literature in this field emphasize the significant role of using normative data specific to a particular population in research or clinical settings.

## Introduction

Hands structure provides individuals with the required grip strength to perform both precise and coarse movement during activities of daily livings (ADLs). Hand function is the outcome of physiologic maturation, neurologic development, and learning patterns associated with functional movements [1]. Children with any neurological or physical hand impairment may be subjected to a reduction of hand strength which will, in turn, jeopardizes their hands’ physical dexterity and functionality, limit their capacity to perform any ADLs.

Grip strength is known as a simple, fast, reliable, and non-expensive index to evaluate the functionality of the upper extremity [2–5]. Moreover, grip strength has been considered as a noninvasive marker of overall muscular strength, muscle mass, nutritional status, and a predictor of cardiovascular and non-cardiovascular mortality, disability, and surgical complications [6–9]. Studies have shown that low handgrip strength (HGS) in children and adolescents is the sign of a poorer metabolic profile and nutritional status which on many occasions will result in obesity, low levels of fitness, and premature mortality in adulthood [10–12]. As a physiological variable, handgrip strength is affected by anthropometric and demographic factors including gender, age, nutritional status, position and orientation of the hand, body size, and hand preference [13–16]. Although some studies have also demonstrated that weight and height are positively correlated with hand strength in pubertal years, the impact of these variables is considerably lesser than that of either gender or age [17, 18]. HGS among adolescents has been reported in the literature with boys having a stronger handgrip strength compared to girls. A similar trend was measured for both girls and boys during puberty [18, 19]. More precisely, the handgrip strength of both genders starts to grow from childhood and it reaches a maximum level at the age of 30s and decreases afterward [20, 21]. Similarly, previous studies on the handgrip strength of children and adolescents show a lower HGS for non-dominant hand in comparison with the dominant one [22, 23].

For clinical evaluation purposes such as identifying the degree of disability and developmental skills level, assessing the integrity of upper limb functions, and determining the efficacy of rehabilitation, the measured handgrip strength of patients is compared with the valid normative data [19, 24, 25]. Ideally, to guarantee a reliable normative handgrip strength data set for the young population, it is necessary to have a large pool of randomly participated subjects to present heterogeneity of the population [26]. Numerous studies have reported normative handgrip strength values for the adults and elderly population [14, 27–30], however, few studies have focused on providing handgrip strength and normative data for children and adolescents. For instance, normative handgrip strength values were reported for adolescents of Saudi Arabia [23], Chile [31], Brazil [32], and some other western countries [33–36]. Some previous studies have revealed that handgrip strength differs among children and adolescents across different regions [35, 37]. These differences can originally be contributed to different ethnicities, nutrition status, sociocultural factors, and leisure activities that affect the variations of the skeletal muscle mass [38, 39].

Although studies in different countries have presented the normative handgrip data for children and adolescents to determine the progress of different disorders and suggest an evidence-based treatment, there is no study on normative handgrip strength data and associated anthropometric/demographic factors on Iranian healthy children and adolescents. Therefore, describing the normative data of handgrip in Iranian healthy boys and girls between 7 and 18 years is important for prioritizing preventive measures in public health efforts and providing a benchmark for assessment of hand function among the aforementioned age group. Another purpose of the current study was to determine the pattern of fluctuations in the handgrip strength among the studied group and to compare it with some other nations.

## Material and methods

### Subjects and sampling

A cross-sectional study of muscle strength was conducted on 2637 healthy children and adolescents, comprising 1391 boys and 1246 girls between the age of 7 and 18 years. Data collection was carried out between February and May 2019.

The three-stage sampling method was utilized. At first, a cluster sampling method was used to identify 10 clusters based on population distribution in Tehran. In the second stage, after providing the list of all the schools located in selected clusters, a systematic random sampling method was applied to choose four schools per cluster (one elementary and one high school for each gender). The required minimum sample size at any of the girls’ or boys’ schools was estimated using Eq. (1) given in “General requirements for establishing anthropometric databases” [40]. The 95% confidence interval was used for the 50th percentile or average values:

1 $$ \mathrm{n}\ge {\left(3.006\times \frac{\mathrm{CV}}{\upalpha}\right)}^2\kern0.5em \mathrm{and}\kern0.75em \mathrm{CV}=\frac{S}{\overline{X}}\times 100 $$

Where n, CV, and α represent the sample size, coefficient of variation, and percentage of the desired relative accuracy, respectively. Assuming a relative accuracy of 5% and using the empirical means and standard deviations (boys: 22.8 kg and 2.9 kg with CV = 12.7; girls: 17.4 kg and 2.3 kg with CV = 13.2) from the results of the initial pilot study of 80 participants (40 for each gender), the required minimum sample size was calculated as 58 for boys and 63 for girls in each school. Considering the “Design effect” for the clustered sampling method (Deff = 2.2) [41], the desired sample size worked out to be 2637 subjects with about 10% allowable error and non-response rate.

All adolescents over 16 years and the parents/guardians of all minor participants (< 16 years) were informed about the study and gave their written consent to participate. Using a short health screening questionnaire, students with a history of fracture, deformity, or surgery in upper extremities during the past year as well as those with a history of specific diseases such as rheumatic arthritis, osteoarthritis, coronary heart disease, chronic kidney disease, and liver cirrhosis were excluded. The impact of these diseases on upper extremities function, especially the arms and hands, has been shown in previous studies [42, 43]. The study was conducted according to the World Medical Association Declaration of Helsinki and was approved by the ethics committee, Iran University of medical science (IR.IUMS.REC 1396.32516).

### Measurements procedures

All measurements were obtained by a trained examiner in a separate room dedicated to the school health supervisor during the school day from 8 to 12 AM. The age of each student was calculated based on the date of birth recorded in his/her educational record. Body mass was measured using a digital balance (Toledo, Model 2096PP/2, Inc., Brazil) to the nearest 0.1 kg. Stature was measured for each subject using the Holtain Harpenden stadiometer (Holtain, Crosswell, UK) to the nearest ±0.1 cm. Body mass index was calculated in kg/m^2^.

**Fig. 1:**
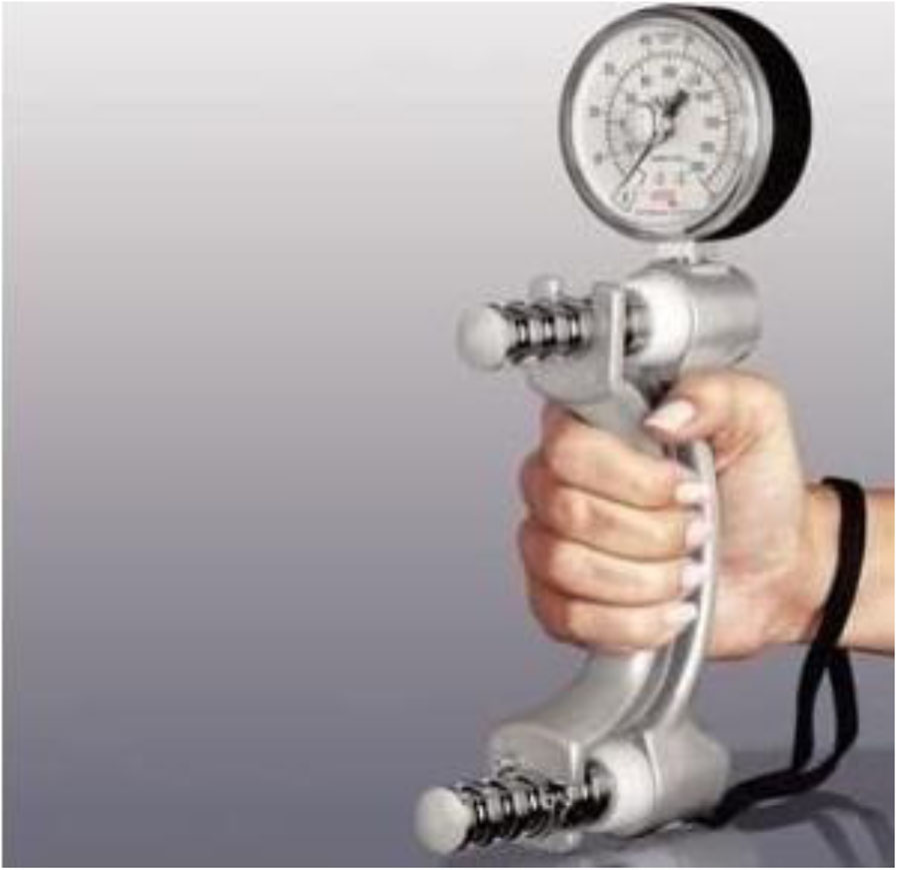
Jamar hydraulic dynamometer (Model 5030 J1, Sammons Preston Rolyan, Bolingbrook, IL, USA)

Handgrip strength was measured with a standard adjustable Jamar hydraulic dynamometer (Model 5030 J1, Sammons Preston Rolyan, Bolingbrook, IL, USA))Fig. 1) based on the recommendation of the American Society of Hand Therapists (ASHT) [24, 44]. For standardization, the dynamometer was set at the second handle position for the measurement of handgrip strength [29]. Students were in comfortable clothing allowing them free arm and hand movement. Hand watch or jewelry were removed from both upper extremities. Before starting the test, hand dominance was determined by asking: “Which hand do you write with? “. Handgrip strength was measured while students were seated with hips and knees flexed at 90^o^ and feet flat on the floor, arms hanging relaxed at the side and neutrally rotated, elbows flexed 90 degrees, and forearm and wrist in neutral position (0–15 degrees of extension and 0–15 degrees of ulnar deviation) [45, 46]. During data collection, the forearm and arm were not supported by the examiner or by an armrest. Students were asked to squeeze the handle of the dynamometer as hard as they could. Verbal encouragement (i.e. squeeze as hard as you can) was provided to ensure maximal effort during each measurement. For each hand, three trials were recorded and the average of the three values was considered as the HGS values for subsequent analyses. If one of the measurements had a difference higher than 10% compared to other measurements, it was cancelled and replaced by a fourth measurement. One-minute rests were given between each attempt to minimize fatigue effects. These procedures have been previously well documented [47, 48]. The calibration of instruments was periodically performed during the study according to the manufacturer’s manual. Also, the dynamometer was set to zero kg before each measurement. Figure 1 shows the dynamometer used for the explained procedure.

### International norms

Normative data obtained from the present study were compared to those previously reported for the sample population of the USA [49], China [29], Norway [35], Sweden [50], Chile [31], Saudi Arabia [23], and Germany [36].

### Data analysis

Statistical analysis was performed by SPSS 23 (IBM Corporation, New York, NY, United States). The normality test was carried out using the Kolmogorov-Smirnov test and confirmed for all data sets. Statistical outliers were checked using Grubb′s test that is based on the difference of the mean of the sample and the most extreme data considering the standard deviation [51]. An independent sample *t-test* was carried out to determine the HGS differences between boys and girls. Paired t-tests were performed to compare the handgrip strength of the dominant hand vs the non-dominant one. The variations of HGS in various ages were tested by one-way ANOVA with Scheffe’s posthoc contrast. Analysis of covariance (ANCOVAs) was used to determine the effect of age, gender, and age/gender combined on handgrip strength. All assumptions regarding ANCOVAs were checked and upheld before performing data analysis. The values of *p* < 0.05 were considered statistically significant.

## Results

### Demographic characteristics

Demographic information including age, gender, and hand dominance of study participants are shown in Table 1. The mean and standard deviation of the age for boys and girls was 13.2 ± 3.7 years and 12.6 ± 2.9 years, respectively. The study sample included 2637 healthy children and adolescents aged 7–18 years: 1391 (52.7%) were boys and 1246 (47.3%) were girls. Right-hand dominance was reported by 2506 (95%) students comprising 1319 (50%) boys and 1187 (45%) girls. None of the students reported ambidexterity.

**Table 1:** Characteristics of study participants: age, gender, and hand dominance

Age (years)	N	Boys			Girls		
		N	Dominant Hand		n	Dominant Hand	
			Right	Left		Right	Left
7	230	126	121	5	104	100	4
8	213	110	103	7	103	98	5
9	235	125	121	4	110	105	5
10	223	116	111	5	107	100	7
11	208	108	105	3	100	96	4
12	212	116	110	6	96	92	4
13	218	114	107	7	104	103	1
14	218	107	103	4	111	105	6
15	208	109	102	7	99	95	4
16	226	118	110	8	108	104	4
17	224	120	111	9	104	96	8
18	222	122	115	7	100	93	7
**Total**	**2637**	**1391**	**1319**	**72**	**1246**	**1187**	**59**

N: number of participants per age

n: number of participants per gender

### Normative values on HGS

Normative handgrip strength data of Iranian healthy children and adolescents are presented in Table 2.

**Table 2:** Descriptive statistics for handgrip strength (kg) stratified by age, hand dominance, and gender

Age (years)	Hand	Mean ± SD (Min-Max)	
		Boys	Girls
7	D	9.9 ± 2.48 (7.1–13.9)	8.4 ± 2.21 (6.1–13.8)
	ND	9.1 ± 2.61 (6.6–12.8)	7.5 ± 2.32 (5.3–12.9)
8	D	11.6 ± 2.51 (8.3–14.6)	9.8 ± 3.10 (7.6–15.1)
	ND	10.8 ± 2.93 (8.4–13.7)	8.9 ± 3.21 (6.9–15.2)
9	D	13.7 ± 3.82 (11.2–17.9)	11.6 ± 3.21 (8.4–16.3)
	ND	12.4 ± 3.61 (10.8–17.2)	10.5 ± 3.73 (8.2–15.7)
10	D	15.9 ± 2.87 (12.7–19.3)	13.4 ± 3.31 (10.7–18.4)
	ND	14.4 ± 2.35 (12.1–18.7)	12.2 ± 2.92 (10.6–17.8)
11	D	18.4 ± 4.15 (13.4–25.2)	16.1 ± 3.61 (12.7–20.1)
	ND	16.8 ± 3.95 (13.0–23.6)	14.6 ± 4.12 (13.0–19.4)
12	D	21.5 ± 4.07 (16.7–26.9)	18.0 ± 3.73 (14.3–20.6)
	ND	19.7 ± 4.25 (15.8–24.7)	16.4 ± 3.27 (15.0–21.2)
13	D	25.8 ± 4.42 (19.8–30.9)	20.0 ± 3.66 (16.6–25.7)
	ND	24.1 ± 4.37 (20.1–31.0)	19.2 ± 3.37 (16.0–24.3)
14	D	30.6 ± 4.34 (24.2–37.0)	21.5 ± 3.82 (16.8–27.5)
	ND	28.8 ± 4.13 (23.4–33.7)	21.1 ± 3.79 (16.6–29.2)
15	D	35.1 ± 4.73 (29.3–40.6)	22.8 ± 3.77 (17.7–26.7)
	ND	33.1 ± 4.28 (27.3–38.5)	21.7 ± 3.47 (16.5–28.1)
16	D	38.4 ± 3.79 (32.0–42.8)	23.7 ± 3.45 (20.1–28.0)
	ND	35.7 ± 3.61 (29.2–40.2)	22.6 ± 3.51 (19.4–28.6)
17	D	41.6 ± 4.13 (35.6–48.7)	24.0 ± 3.20 (19.7–29.0)
	ND	38.6 ± 4.27 (33.4–45.1)	22.5 ± 3.25 (19.0–28.4)
18	D	42.8 ± 4.11 (33.6–47.6)	24.3 ± 3.71 (20.0–30.4)
	ND	39.6 ± 4.55 (34.8–44.5)	22.7 ± 3.60 (18.6–29.8)
**Total**	**D**	**25.6 ± 3.85 (7.1–48.7)**	**17.8 ± 3.27 (6.1–30.4)**
	**ND**	**23.3 ± 4.10 (6.6–45.1)**	**16.6 ± 3.12 (5.3–29.8)**

D: Dominant hand, ND: Non-dominant hand

### The effect of age, gender, and hand dominance on HGS

Age, gender, and their interaction effects on handgrip strength were studied by ANCOVAs (Table 3). According to this, age significantly affected dominant and non-dominant handgrip strength measurements (F = 4217.83 and F = 4028.52 for dominant and non-dominant HGS, respectively; *P* < 0.0001). The effect of gender on handgrip strength was also meaningful (F = 131.21 and F = 128.33 for dominant and non-dominant HGS, respectively; *p* < 0.0001); boys had a greater handgrip strength than the girls at all ages. More precisely, the average of girls’ dominant and non-dominant HGS was approximately 56 and 59% of boys, respectively. The age and gender interaction effects on HGS were also significant (F = 354.67 and F = 349.12 for dominant and non-dominant HGS, respectively; p < 0.0001). Hand dominance also had a significant effect on grip strength (*p* < 0.001). So, the dominant hand was stronger than the non-dominant hand by about 6–10% and 2–10% for boys and girls, respectively.

**Table 3:** Univariate (unadjusted) ANCOVAs results for age, gender, and age/gender

Variable	F_1,2636_ Values		
	Age	Gender	Age/ Gender
HGS_D_	4217.83	131.21	354.67
HGS_ND_	4028.52	128.33	349.12

HGS_**D**_: Dominant handgrip strength

HGS_**ND**_: Non-dominant handgrip strength

Note: All *p-*values were < .0001

### Fluctuation patterns and comparison with other nations’ norms

Mean handgrip strength showed an increasing curve in boys (slope ≈ up to 27%, *p* < 0.001) and girls (slope ≈ up to 22%, *p* < 0.001). For 7 to 13 years old boys, a significant difference was found in handgrip strength per year relative to the last year with an ascending trend (p < 0.001). Thereafter, the difference shows a significant and decreasing trend relative to the previous year until the age of 17 years (*p* < 0.001). However, there was no significant difference between the handgrip strength of boys from 17 to 18 years (less than 2.5%, *p* > 0.05). Among girls aged 7 to 11 years, a significant difference was discerned in handgrip strength per year relative to the previous year with a decreasing trend for thereafter years until the age of 14 years old (p < 0.001). However, the annual increase of handgrip strength was negligible for girls over the age of 14 years (*p* > 0.001).

**Fig. 2:**
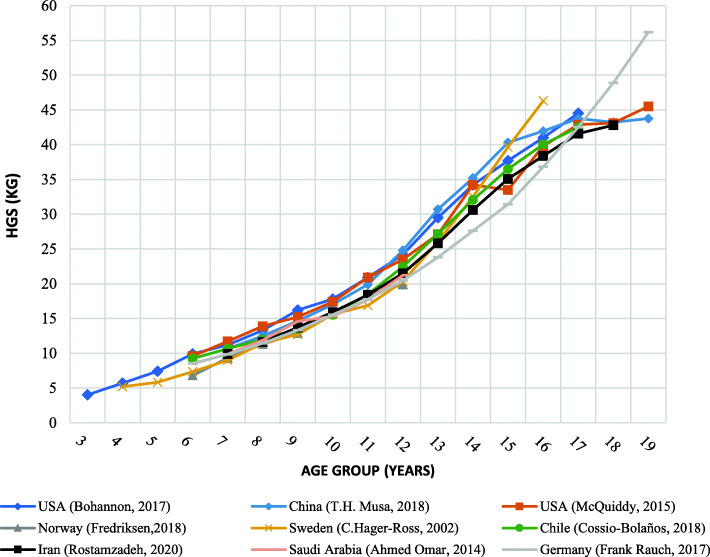
Boys’ regional reports of mean handgrip strength for a different age

**Fig. 3:**
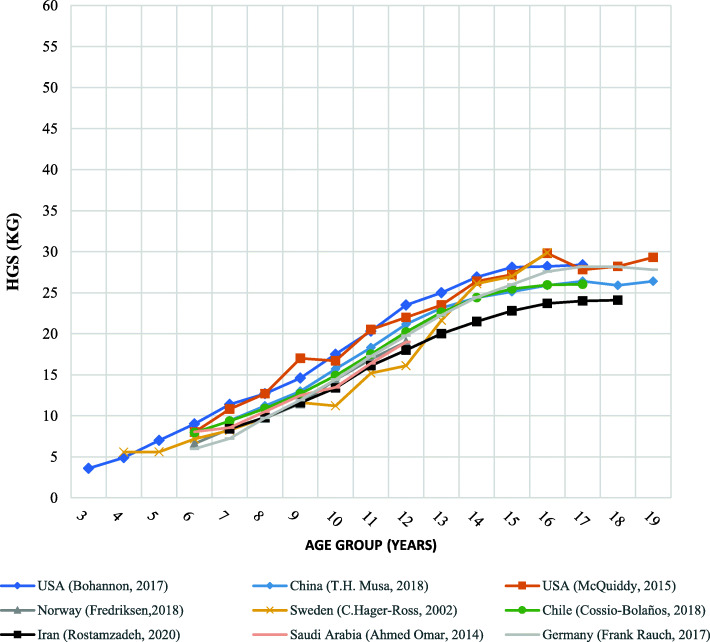
Girls’ regional reports of mean handgrip strength for a different age

Fluctuations of grip force across the childhood and adolescence of different nations are presented in Figs. 2 and 3. The findings of our study showed no significant difference between the handgrip strength of the Iranian boys and those of the population’s norms in the age range of 7–11 years (p > 0.001). However, the handgrip strength of the Iranian boys in the age range of 12–17 years is lower than the American (by about 6–12%) and Chinese (by about 5–16%) counterparts. The highest difference of the handgrip strength between Iranian boys with American and Chinese boys is observed at the age of 14 years (*p* < 0.001). Comparing our results with those of other nations suggested that peak values of HGS reached in Iranian school-aged boys at the age of 18 years (42.8 kg for the dominant hand and 39.6 kg for non-dominant hands) are lower than those reached in the German population (48.9 kg for the dominant hand and 45.9 kg for non-dominant hand), but almost close to those reached in Chinese (43.2 kg for the dominant hand and 40.3 kg for non-dominant hand) and American (43.1 kg for the dominant hand and 40.6 kg for non-dominant hand). However, the fast rise of handgrip strength in Swedish and German boys over the age of 12 years is notable, and the handgrip strength of Iranian boys in this age range grows with a moderate slope than that Swedish and German boys (with an average slope of 12% for Iranian boys compared to an average slope of 17 and 23% for German and Swedish boys, respectively).

A similar pattern can be seen for Iranian healthy girls in “Fig. 3”. Their grip strength is lower than Americans (by about 15–30%, where the highest difference is observed in the aged 7 and 9 years), Chilean (by about 8–12%, where the highest difference is observed in the aged 14 years), and Chinese (by about 7–15%, where the highest difference is observed in the aged 12 years) girls. Only in the 9–12 years age range, the mean HGS of Iranian girls was slightly higher than that of Swedish girls, except for the narrow differences that were observed at the age of 11 years. Over the age of 13 years, the handgrip strength of Iranian school-aged girls was considerably lower than German and Swedish girls. While before the age range, there was no significant difference between the handgrip strength of the Iranian girls and these two populations (*p* > 0.001). As seen in “Fig. 3”, differences in handgrip strength between Iranian girls with those living in Saudi Arabia, German, and Norway appeared to be rather small in the age range of 7–12 years (p > 0.001).

## Discussion

The present study established reference values of handgrip strength for Iranian healthy children and adolescents, aged 7–18 years old. The study was not only able to develop a valid and reliable benchmark for determining the effectiveness of clinical treatments, but also a comparable dataset with other international norms.

Overall patterns of handgrip strength associated with age in both genders were comparable to those of previous studies. This pattern of progressive strength may be explained by the similar arm and forearm muscle group development that is generally independent of the geographical area among the boys and girls up to age 16 across the world [49, 50, 52, 53]. The processes of growing up of boys and girls stem from several factors such as the mechanical stress leading to the increase of the body weight, proper biomechanical cues to the processes of growing long bones, a rise in androgen hormones of both genders in pubertal years, and possibly, the direct impact of adrenal and gender steroids on the muscle [54, 55]. The hormonal impact on skeletal muscle mass and a study of muscle progression in healthy children and adolescents indicates a necessity to relate the action of estrogen, testosterone, and growth hormone in the development of muscles during puberty and the early post-pubertal [56, 57]. Handgrip strength showed a linear and parallel development for boys and girls until the age of 11 years, after which handgrip strength progression shows steeper upward slope in boys than the girls, which was similar to the findings of Ahmed Omar et al. [23] and Hager-Ross et al. [50]. Concerning gender, our results were similar to the several studies which have shown that HGS is higher in boys than in girls [17, 23, 34]. Some studies have suggested that an increase in boys’ testosterone during puberty is a major factor in their increasing muscle strength [58]. The faster growth rate of the musculoskeletal structure of the arm and hands in males may be due to a larger amount of testosterone in boys’ bloodstream which in turn results in a greater muscle density of males than the females [59, 60]. Boys are said to be able to exert a larger body strength in comparison to girls of the same age, this may be explained by the fact that boys are known to have motoneuron adaptation to recruit a larger muscle fiber to perform any ADLs [61]. In the present study, handgrip strength was affirmed to be significantly greater in the dominant hand compared to the non-dominant one. This finding can be emphasized by other studies on HGS [18, 62]. Sartorio et al. showed that the hand strength of the dominant hand was 10% greater compared with the non-dominant for all boys and girls [63]. Our measurement showed that the difference in the grip strength between dominant and non-dominant hands was about 8% for both genders. The study by Go’mez-Campos et al. [31] in Chile, where the authors evaluated the same age bracket, showed that the mean values of dominant handgrip strength were slightly higher than the non-dominant side. Main reason for the differences between two studies may be explained by authors hand group classification in which they simply grouped the right- and left hand instead of considering a specific variable to focus on a dominant and non- dominant hand.

The findings of this study showed the handgrip strength between Iranian children/adolescents and other international studies was having a similar upward trend as the other nations who have already reported in the literature. In 2017, Bohannon and colleagues reported handgrip strength norms versus age as an independent variable for those between 3- to 17-year-old Americans [49]. There was no significant difference in the trend of the handgrip strength of the Iranian and American boys in the age range of 7–11 years. However, the HGS of Iranian boys over the age of 12 years were slightly weaker than the American and Chinese counterparts meaning that grip strength was measured and plotted with the flatter slope than American and Chinese [36, 50]. The rapid growth of the body sizes and muscle mass, regular physical and sports activities, and proper nutrition before pubertal years may be the main reasons for the muscle growth in American and Chinese boys over the age of 12 years [64]. Our results showed that the HGS of Iranian girls were considerably lower than American, Chilean, and Chinese peoples by average 23, 10, and 11%, respectively. These differences are likely due to the different ethnic and geographic pools from which the populations were drawn. Additionally, variations in grip strength norms from different regions and nations are believed to be largely dependent on the hand size and anthropometric differences [14, 30, 65, 66]. Moreover, Iranian girls under the age of 12 had the handgrip strength almost equal to those of German and Swedish girls. However, Iranian girls were exerting considerably weaker grip strength than German and Swedish girls after the age of 12 years. This finding may be attributed to the earlier malnutrition of the children during infancy by which bone density and mineralization are minimized resulting in less development of muscular mass during adolescence and after the age of 11 years, therefore, less grip strength may be produced is observed [67]. Our findings recommend that grip strength norms from the Western and European populations may not reflect precisely the indigenous population living in Iran due to different trend lines; therefore native reference values are required [26, 30].

The strength of this study has relied on the following factors; first and foremost, it was performed by considering a large sample of children and adolescents. Second, standard protocols were considered for the assessment of the handgrip strength and data monitoring processes (data collection, data entry, and data analysis). The current investigation is limited because the study is a cross-sectional analysis; thus, the presence or absence of a causal relationship cannot be determined. Additionally, several variables such as nutritional status, occupation, physical activity, and maturity stages were not considered, which can be regarded as a reasonable limitation due to the potential for misinterpretation.

## Conclusions

The results of this study provide baseline information about handgrip strength norms in Iranian children/adolescents. The trend of the findings had a similar pattern to other countries that have already reported a similar study. Both Iranian girls from age of 7 to 18 and boys from 12 to 18 were having an HGS that was quite weaker than their western counterparts. The differences may be underlying many factors including hormonal, nutritional variations, as well as sociodemographic and anthropometric difference s. In addition, the results from this study showed that gender, age, and hand preference can be influential variables on the development of handgrip strength. The data reported will enable therapists and pediatric clinicians to compare a patients’ grip strength measurements with normative values according to age, gender, and dominance. The illustrated results may be useful for ergonomists in the design of comfortable hand tools and stationeries for children and adolescents.

## Data Availability

Data are not publicly available. These study data were not anonymous. Due to the sensitive nature of the data and privacy and confidentiality guidelines, the data must be housed in a secured lab and cannot be made publicly available.
